# Benzo(a)pyrene triggers desensitization of β2-adrenergic pathway

**DOI:** 10.1038/s41598-017-03646-4

**Published:** 2017-06-12

**Authors:** Abdullah Mayati, Normand Podechard, Manuelle Rineau, Lydie Sparfel, Dominique Lagadic-Gossmann, Olivier Fardel, Eric Le Ferrec

**Affiliations:** 1 Inserm U1085, Institut de Recherche en Santé, Environnement, Travail, Rennes France; 2 Université de Rennes 1, Faculté des Sciences pharmaceutiques et biologiques, Biosit UMS3080, 35043 Rennes Cédex, France; 3 Pôle Biologie, Centre Hospitalier Universitaire, 2 rue Henri Le Guilloux, 35033 Rennes, France

## Abstract

Exposure to environmental polycyclic aromatic hydrocarbons (PAHs), such as benzo(a)pyrene (B(a)P), has been linked to several health-threatening risks. PAHs were also shown to hinder adrenergic receptor (ADR) responses. As we previously demonstrated that B(a)P can directly interact with the β2ADR, we investigated here whether B(a)P could decrease β2ADR responsiveness by triggering receptor desensitization phenomena. We firstly showed that exposure to B(a)P reduced β2ADR-mediated epinephrine-induced induction of NR4A gene mRNAs and of intracellular cAMP. Analysis of β2ADR protein expression demonstrated that B(a)P rapidly decreased membrane expression of β2ADR with a subsequent degradation of receptor protein. B(a)P exposure concomitantly rapidly increased the β2ADR mRNA levels. The use of the β-blockers, propranolol and ICI 118.551, demonstrated the involvement of β2ADR itself in this increase. However, sustained exposure to B(a)P induced a diminution of β2ADR mRNA steady-state as a result of the acceleration of its degradation. Together, these results show that, beside the well-known activation of the aryl hydrocarbon receptor, PAH deleterious effects may involve the dysfunction of adrenergic responses through, in part, the desensitization of β2ADR. This may be taken in consideration when β2-agonists/antagonists are administered in patients exposed to important concentrations of PAHs, *e.g*. in cigarette smokers.

## Introduction

Polycyclic aromatic hydrocarbons (PAHs), such as the prototypical molecule benzo(a)pyrene (B(a)P), constitute an ubiquitous family of environmental contaminants, that are notably found in cigarette smoke, exhaust particles, grilled foods and industrial waste by-products^1, 2^. Exposure to these pollutants has been correlated to various pathological situations, including inflammation, cancer development, cardiovascular and pulmonary diseases^3, 4^. Therefore, PAHs have been classified as priority toxicants by the United States Environmental Protection Agency (US-EPA), the World Health Organization (WHO) and the European Union^5^. Most of the PAH-related deleterious effects are mediated by activation of the cytosolic aryl hydrocarbon receptor (AhR), and its subsequent binding to specific xenobiotic responsive elements which are found in the promoter of PAH-responsive genes^6^. However, some PAH-related effects may be initiated in an AhR-independent manner, *e.g*. the transient increase in intracellular calcium concentration by B(a)P^7^ and the induction of CXCL8 by nitro-PAHs^8, 9^. Our attempts to explain these AhR-independent effects have recently revealed an important role of β2-adrenergic receptor (β2ADR) signaling pathway^10, 11^. Interestingly, this role seems to involve a direct activation of β2ADR by PAHs, thus adding these pollutants to the list of β2ADR-interacting agents^10^.

β2ADR is a G-protein coupled receptor (GPCR), that is originally activated by catecholamines, *i. e*. adrenaline or epinephrine (EN) and noradrenaline or norepinephrine, resulting in the regulation of crucial physiological functions, including the control of smooth muscle contraction, blood pressure, bronchodilation, and of glycogenolysis^12, 13^. Upon stimulation, β2ADR activates the adenylyl cyclase (AC) *via* a Gs protein, leading to production of cAMP from ATP and activation of downstream effectors, such as protein kinase A (PKA) and exchange protein factor directly activated by cAMP (EPAC)^14^. In parallel to the activation of these signaling pathways, exposure to β2-agonists leads to receptor desensitization; *i.e*. agonist-induced time-dependent loss of β2ADR responsiveness. This negative feedback loop is a multi-step process that results in the reduction of receptor number at both the surface and the inner of the cell by (i) the degradation of the receptor itself after its internalization, and (ii) the decrease of its synthesis *via* the acceleration of mRNA decay^15^.

Interestingly, exposure to PAHs, or to PAH-related pollutants such as the carcinogenic halogenated 2,3,7,8-tetrachlorodibenzo-para-dioxin (TCDD), has been correlated to desensitization-like effects on β2ADR. Indeed, exposure to some environmentally relevant PAHs impaired β2ADR-mediated airway relaxation in human, resulting in a reduced responsiveness to standard therapy of asthma^16^. Moreover exposure to TCDD decreased β-adrenergic responsiveness in chick embryo cardiac muscle cells^17^. These observations highlight the impact of PAH exposure on β2ADR responsiveness. However, to date, understanding of the underlying molecular mechanisms is still limited.

The present study was thus designed to investigate the effect of B(a)P exposure on β2ADR responsiveness to EN in a human cardiovascular cell model, and to verify whether this effect was associated to β2ADR desensitization through focusing on the related cellular mechanisms. Our results revealed that B(a)P decreased the receptor responsiveness to β2-agonists with a concomitant β2ADR internalization and, for more sustained exposures, receptor degradation at both protein and mRNA levels.

## Results

### Validation of cell model for the study of β2ADR activation by EN

**Figure 1:**
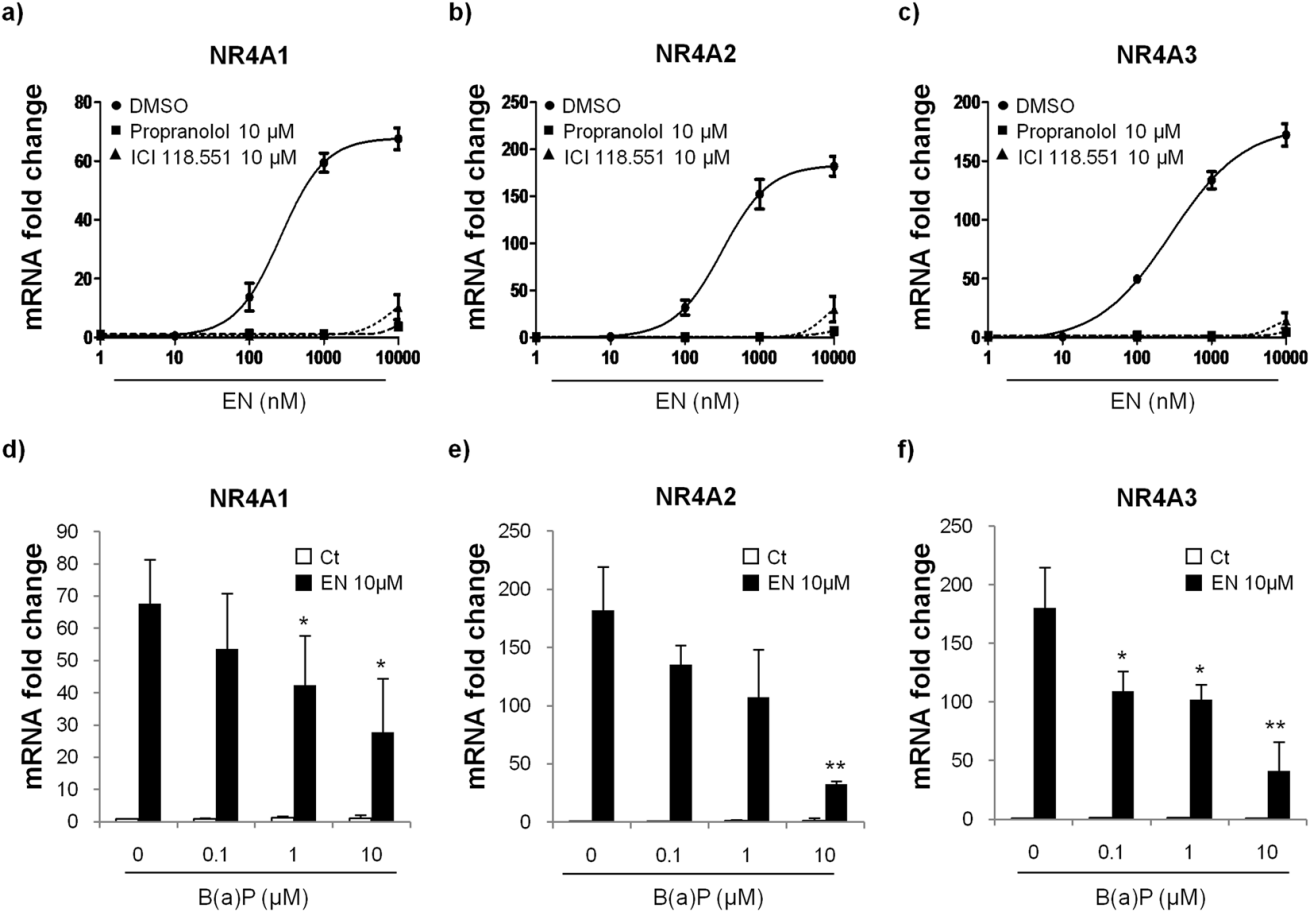
B(a)P decreased β2ADR-mediated induction of NR4As expression triggered by epinephrine (EN). (**a**,**b**,**c**) HMEC-1 cells were either untreated or exposed to indicated concentrations of EN for 1 h, in the presence or not of 10 µM propranolol (nonselective β-blocker) or 10 µM ICI 118.551 (selective β2-bloker). mRNA expressions of β2ADR-target genes, NR4A1 (**a**), NR4A2 (**b**) and NR4A3 (**c**) were next determined by RT-qPCR. (**d**,**e**,**f**) HMEC-1 cells were exposed to indicated concentrations of B(a)P or vehicule (DMSO) for 24 h before being co-exposed, or not, to 10 µM EN for 1 h. mRNA expressions of β2ADR-target genes, NR4A1 (**d**), NR4A2 (**e**) and NR4A3 (**f**) were next determined by RT-qPCR. Data are expressed relatively to mRNA levels found in untreated cells, arbitrarily set to 1 unit, and are the means ± S.D of at least three independent assays. *p < 0.05; **p < 0.01 when compared to B(a)P-untreated cells.

For this work, we used the physiological catecholamine EN as a referent agonist of β2ADR activation. As a study cell model, the human microvascular endothelial cells HMEC-1 were used since they constitute an established model for the study of β2ADR activation^10^, and since they are derived from the cardiovascular system, known to be a major target of PAH toxicity^3^. To validate this choice, the effects of EN on the expression of nuclear receptor 4A (NR4A) mRNAs were evaluated in the presence or absence of β2ADR-antagonists (Fig. 1). Indeed, NR4A subfamily is a group of orphan nuclear receptors, including Nur77 (NR4A1), Nurr1 (NR4A2), and NOR1 (NR4A3), whose expressions are rapidly induced by EN through the activation of β2ADR pathway^18^. As expected, EN induced mRNA expression of NR4A1, 2 and 3 in HMEC-1 cells after 1 h of exposure, in a dose-dependent manner (Fig. 1a,b,c respectively). This effect was strongly hindered by antagonizing β2ADR using the non-selective β-blocker propranolol, or the selective β2-blocker ICI 118.551 (Fig. 1a,b,c), thus demonstrating the involvement of β2ADR in the induction of NR4A genes by EN in our cell model, in accordance with previous reports^19^. This involvement was further consolidated by the ability of the selective β2ADR-agonist salbutamol to increase mRNA level of NR4A family members in a dose-dependent manner (Supplementary Figure S1).

### B(a)P decreased β2ADR-mediated EN-triggered induction of NR4As expression

To test whether B(a)P was able to desensitize β2ADR responsiveness to EN, we evaluated the impact of B(a)P pre-exposure on EN-induced NR4As expressions. HMEC-1 cells were exposed during 24 h to various concentrations of B(a)P (0, 0.1, 1, or 10 µM), and then co-exposed during 1 h to 10 µM EN before analyzing NR4As mRNA levels. As shown in Fig. 1, B(a)P pretreatment, which did not affect cell viability whatever the dose used (Supplementary Figure S2), strongly decreased the EN-induced enhancement of mRNA expression of NR4A1 (Fig. 1d), NR4A2 (Fig. 1e) and NR4A3 (Fig. 1f). In addition, the effect of B(a)P was dose-dependent, starting at 0.1 μM and becoming significant from 1 µM.

### B(a)P pre-treatment decreased EN-induced intracellular cAMP production

**Figure 2:**
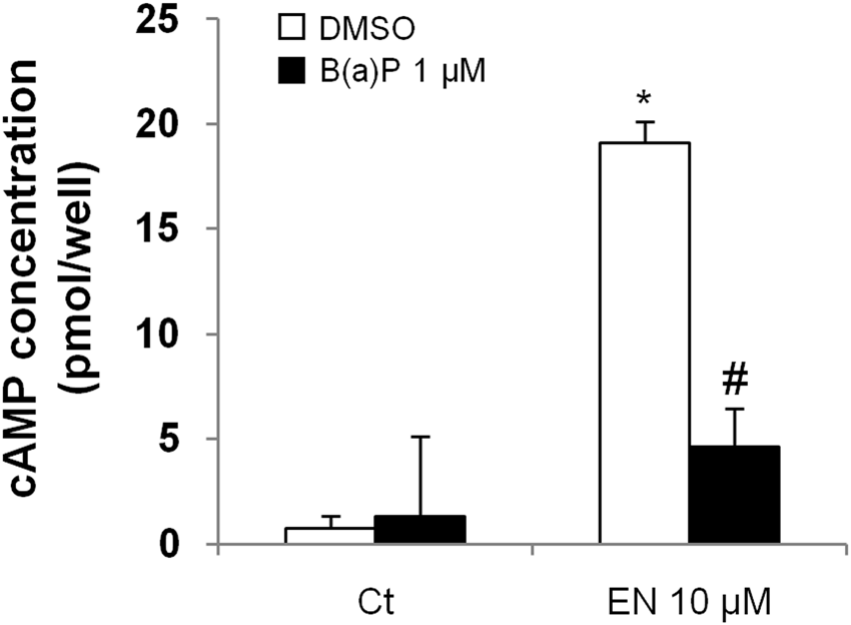
B(a)P pre-treatment decreased EN-induced intracellular cAMP production. HMEC-1 cells were exposed to 10 μM B(a)P or vehicule (DMSO) for 24 h, and were then co-exposed, or not, to 10 µM EN for 10 min. cAMP levels (pmol/well) were next determined, as described in Materials and Methods section. Data are the means ± S.D of three independent assays. *p < 0.05 when compared to untreated cells; ^#^ ***p*** < 0.05 when compared to EN treated counterparts.

As mentioned above, β2ADR is a member of a specific subfamily of GPCRs that is linked by Gs protein to adenylyl cyclase; upon stimulation, this enzyme hydrolyzes ATP and generates the universal second messenger cAMP which can in turn activate downstream effectors^20–22^. Therefore, the increase of cAMP intracellular concentration is widely considered as a hallmark of β2ADR activation. Accordingly, we found that EN rapidly (10 min) and markedly increased cAMP level in HMEC-1 cells (Fig. 2). To further evaluate the influence of B(a)P pretreatment on β2ADR responsiveness to EN, we therefore decided to analyze the effect of EN on intracellular cAMP concentration, with or without prior exposure to B(a)P. Results showed that B(a)P used at 1 µM for 24 h significantly inhibited the EN-induced cAMP increase, thereby confirming our observation on NR4As mRNAs and the ability of B(a)P to decrease cellular responsiveness to stimulation by EN (Fig. 2).

In order to decipher the mechanisms underlying the B(a)P-induced decrease in cell responsiveness to EN, we hypothesized that B(a)P might trigger β2ADR desensitization as a consequence of their direct interaction previously described in HMEC-1 cells^10^. To test this hypothesis, we analyzed the impact of B(a)P exposure on i) the number of available β2ADR at cell surface, ii) the potential degradation of the receptor at protein level, and iii) the reduction of receptor synthesis brought about by the decrease of its mRNA stability.

### Short-term exposure to B(a)P reduced β2ADR density at cell surface

**Figure 3:**
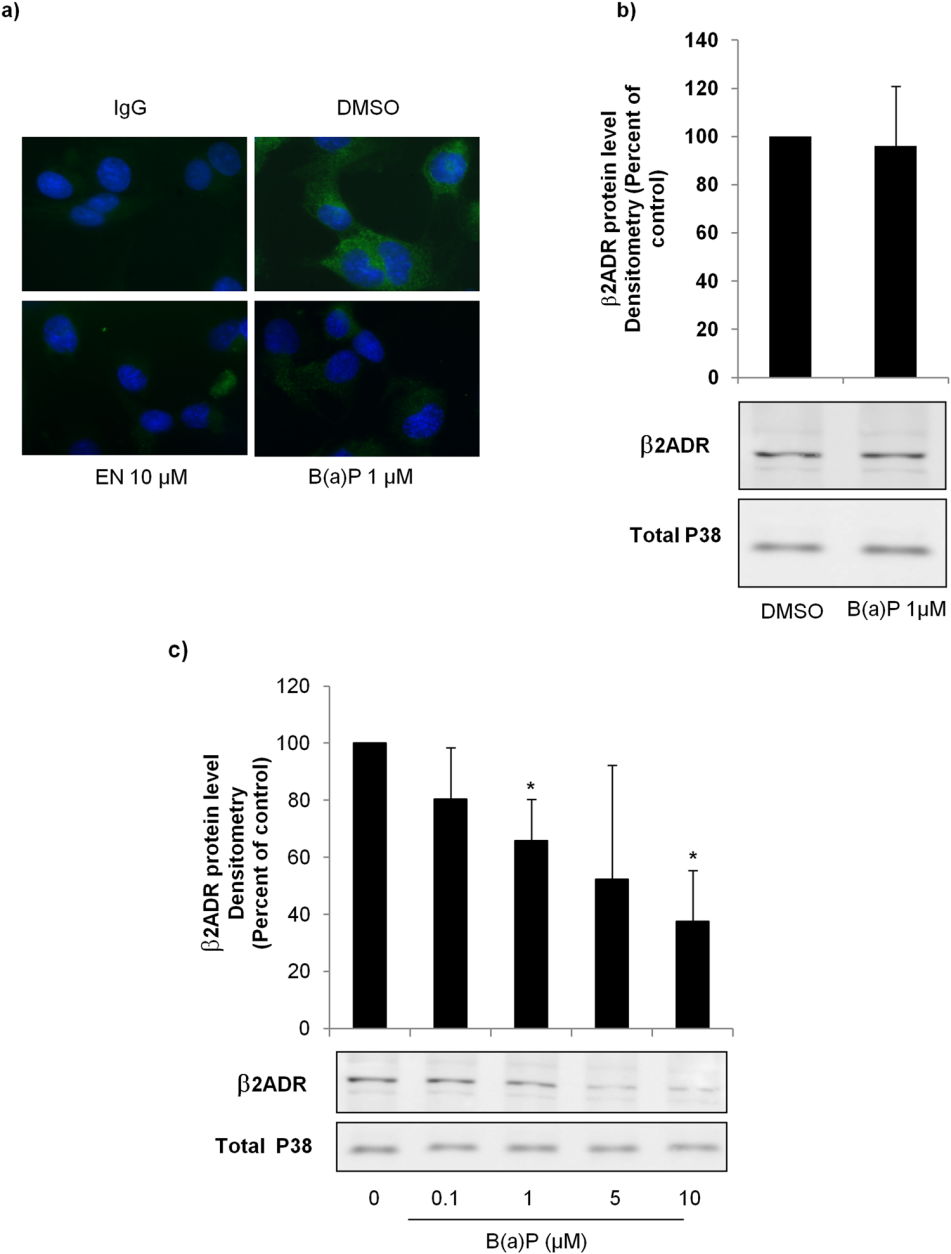
B(a)P triggered an early reduction of β2ADR expression at cell membrane and a late degradation of β2ADR protein. (**a**) HMEC-1 cells were exposed to 10 µM EN, 1 µM B(a)P or vehicle (DMSO) for 45 min. β2ADR was then analyzed by immunolocalization for the expression at cell membrane. Data shown are representative of three independent assays. (**b**) HMEC-1 cells were exposed to 1 µM B(a)P or vehicle (DMSO) for 45 min. β2ADR protein content was then determined by Western-blotting. (**c**) HMEC-1 cells were exposed to indicated concentrations of B(a)P or vehicle (DMSO) for 24 h. β2ADR protein content was then determined by Western-blotting. (**b** and **c**) Bottom panel, a representative blot is shown for β2ADR and P38 protein levels. Upper panel, for each concentration of B(a)P, data were quantified by densitometric analysis, normalized to P38 protein level and expressed relative to β2ADR found in untreated cells, arbitrarily set at the value of 100%. Results are the means ± S.D of values from three independent assays. *p < 0.05 when compared to untreated cells.

Agonist-induced reduction in membrane β2ADR density is usually related to receptor internalization after few minutes of exposure to agonist^23^. To examine the hypothesis that B(a)P might generate such an effect, we performed immunolocalization assays to evaluate the density of β2ADR at the cell surface of HMEC-1 after a 45 min-exposure to 10 µM of EN (used here as a positive control for the activation of β2ADR) or to 1 µM of B(a)P. As previously demonstrated^24^, activation of β2ADR by EN led to a marked reduction in fluorescent signal at the cell surface, thus reflecting a decrease in receptor number (Fig. 3a). Similarly, exposure to B(a)P reduced the β2ADR signal density (Fig. 3a). To consolidate these results, fluorescence signals were quantified (Supplementary Figure S3). To determine whether this decrease was associated or not with protein degradation, we next realized a Western blot analysis of total protein extracts of HMEC-1 cells treated or not with 1 µM of B(a)P for 45 min. As no reduction of β2ADR protein level was detected (Fig. 3b), results of immunolocalization argue in favor of the internalization of β2ADR without any protein degradation at this time point.

### Sustained exposure to B(a)P triggered β2ADR protein degradation

It is well known that the rapid internalization of β2ADR may be followed, upon sustained stimulation, by its downregulation partly due to the degradation of the receptor protein. In order to investigate the potential role of B(a)P in triggering similar effects, cells were exposed during 24 h to different concentrations of B(a)P (0, 0.1, 1, 5, or 10 µM), before studying β2ADR protein levels by Western blotting of total protein extracts. Results shown in Fig. 3c indicated that B(a)P reduced β2ADR protein level. This effect was detected at all tested doses, but was only statistically significant at 1 and 10 µM of B(a)P (Fig. 3c).

### Sustained exposure to B(a)P induced β2ADR mRNA degradation

In addition to the degradation of receptor protein, the agonist-induced down-regulation of β2ADR is also the output of decreased receptor synthesis resulting from the acceleration of its mRNA decay^15^. We therefore decided to analyze the influence of B(a)P exposure on the β2ADR mRNA expression at several time points in HMEC-1 cells. To do so, cells were exposed to 1 µM B(a)P or 10 µM EN (as a positive control for β2ADR activation) during 0, 0.5, 1, 2, 3, 6 or 8 h before measuring β2ADR mRNA levels.

**Figure 4:**
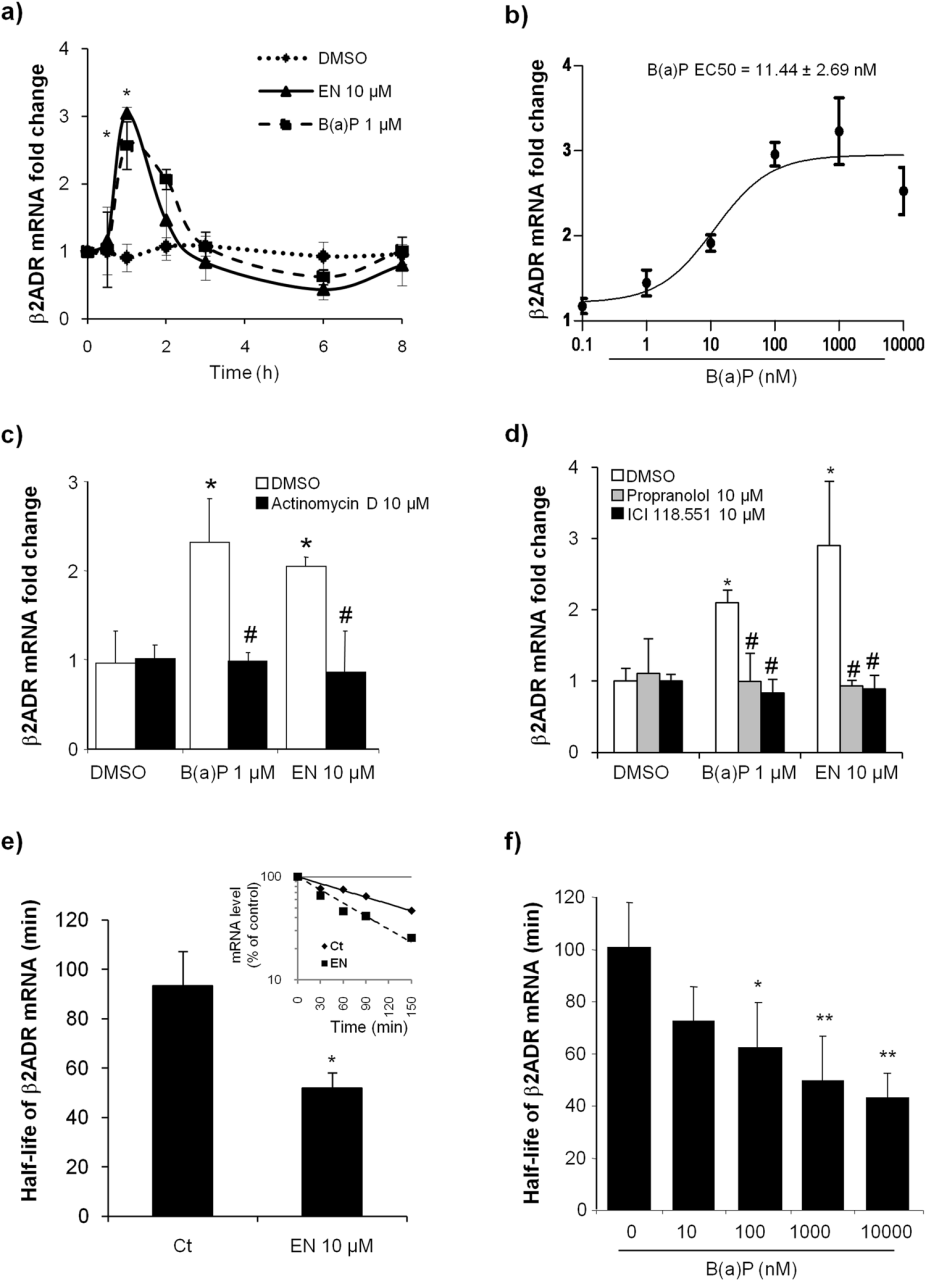
B(a)P exposure influenced β2ADR mRNA level. (**a**) HMEC-1 cells were exposed to 10 µM EN, 1 µM B(a)P or vehicle (DMSO) for indicated times before analyzing β2ADR mRNA expressions by RT-qPCR. Data are expressed relatively to mRNA levels found in corresponding control cells, arbitrarily set to 1 unit, and are the means ± S.D of at least three independent assays. *p < 0.05 when compared to corresponding control cells. (**b**) HMEC-1 cells were exposed to indicated concentrations of B(a)P or vehicle (DMSO) for 1 h. mRNA expressions of β2ADR were then analyzed by RT-qPCR. Data are expressed relatively to mRNA levels found in control cells, arbitrarily set to 1 unit, and are the means ± S.D of at least three independent assays. (**c**,**d**) HMEC-1 cells were exposed to 10 µM EN, 1 µM B(a)P or vehicle (DMSO) for 1 h, in the presence or absence of 10 µM actinomycin D (**c**), or in the presence or absence of 10 µM propranolol or 10 µM ICI 118.551 (**d**). mRNA expressions of β2ADR were then analyzed by RT-qPCR. Data are expressed relatively to mRNA levels found in corresponding control cells, arbitrarily set to 1 unit, and are the means ± S.D of at least three independent assays. *p < 0.05 when compared with conotrol cells. ^*#*^ ***p*** < 0.05 when compared to EN- or B(a)P-treated counterparts. (**e**,**f**) HMEC-1 cells were either untreated or exposed (1 h) to 10 µM EN (**e**), or were exposed to indicated concentrations of B(a)P or vehicle (DMSO) (**f**), Half-lives of β2ADR mRNA were then calculated as described in Materials and Methods section. Data correspond to mRNA half-lives, expressed in minutes, and are the means ± S.D of three independent assays. *p < 0.05 when compared with untreated cells. ^*#*^ ***p*** < 0.05 when compared to EN- or B(a)P-treated counterparts. Representative curves of mRNA decay kinetics in the absence (Ct) or presence of EN are given in the insert (**e**).

Figure 4a demonstrates that B(a)P and EN induced similar biphasic modifications on the mRNA level encoding the β2ADR in our cellular model. Indeed, the addition of either B(a)P or EN rapidly increased the expression of β2ADR mRNA, with a peak at 1 h of exposure. This first phase was then followed, after a more sustained exposure, by a reduction in the steady-state mRNA level, with the lowest level reached after 6 h of treatment (reduction by 50% compared to DMSO control condition); a return to basal levels was then observed at 8 h (Fig. 4a).

To get more insight about the B(a)P-induced biphasic modulations of β2ADR mRNA level, we then separately studied each of these two phases.

Regarding the early transient increase, it was shown to depend on the concentration of B(a)P used at 1 h of exposure. As demonstrated in Fig. 4b, the effect of B(a)P was from 10 nM of B(a)P and maximal at 1 µM (EC50 = 11.44 ± 2.69 nM). In addition, when HMEC-1 cells were pretreated with the transcriptional inhibitor actinomycin D (5 μg/ml) and then stimulated with B(a)P 1 µM or EN 10 µM (used as a positive control of β2ADR activation) for 1 h, mRNA analysis demonstrates that actinomycin D completely abolished the B(a)P and EN induced effect, as shown in Fig. 4c. Furthermore, the non-selective β-blocker propranolol, and the selective β2-blocker ICI 118.551, both inhibited this induction of β2ADR mRNA level observed in presence of B(a)P and EN (Fig. 4d). These results demonstrate the transcriptional nature of the B(a)P-triggered induction of β2ADR mRNA and the involvement of the receptor itself. Taking into account that similar observations were associated to β2-agonist exposure^25^, these results pointed to an agonist-like effect of B(a)P on β2ADR.

With regard to the reduction of β2ADR transcript level, it has been suggested that β2-agonists may reduce receptor mRNA stability^15^. We thus analyzed the influence of B(a)P on the half-life of β2ADR mRNA, as described in Materials and Methods section. Under control conditions, results demonstrated that in our cell model and culture conditions the half-life of β2ADR mRNA was 91.2 ± 13.3 min, in accordance with the literature^15^. In presence of EN, this half-life value was reduced to 50.6 ± 6.6 min (Fig. 4e), thus indicating an accelerated rate of decay of β2ADR mRNA upon receptor activation. Under the same experimental conditions, we determined β2ADR mRNA half-lives in the presence of various concentrations of B(a)P, *i.e*., 0, 10, 100, 1000, or 10000 nM. Our results indicated that B(a)P exposure elicited a dose-dependent reduction of β2ADR mRNA half-life (Fig. 4f). This effect was noticeable at 10 nM and significant from 100 nM of B(a)P.

## Discussion

We previously demonstrated that the prototypical environmental pollutant B(a)P can directly bind to β2ADR to activate the adenylyl cyclase/cAMP/Epac-1/inositol 1,4,5-trisphosphate/Ca^2+^ pathway^10^. While additional studies are needed, those results have suggested that B(a)P could behave like a β2ADR agonist. Since it is well documented that activation of β2ADR initiates a desensitization of the receptor^26^, the focus was herein on putative β2ADR desensitization phenomena triggered by B(a)P. Our results indicate that the referent PAH is able to markedly reduce HMEC-1 cells’ responsiveness to β2ADR activation by β2-agonists (*e.g*. EN), as a consequence of receptor desensitization, *i.e*. receptor internalization followed by receptor mRNA and protein degradation. These results are the first demonstration, to the best of our knowledge, of the molecular mechanisms of the previously documented β2ADR loss-of-function after exposure to PAH and PAH-related contaminants^16, 27^.

The decrease of β2ADR responsiveness to EN after exposure to PAHs was supported by the capacity of B(a)P (i) to inhibit EN-triggered β2ADR-mediated induction of NR4A1, 2 and 3 expressions and (ii) to reduce the cAMP signal induced by EN. It is noteworthy that this loss of responsiveness to EN was detected at relatively low concentrations of B(a)P (Figs 1d,e,f, 3c and 4f). Even if these concentrations of B(a)P remain higher than those to whom humans are exposed^28^, one might speculate that synergic effects of various B(a)P-related PAHs, which exist as mixtures in the environment and in cigarette smoke, might contribute to β2ADR loss-of-function at more environmentally relevant concentrations. This hypothesis may be supported by (i) the capacity of other PAHs, such as pyrene (10 µM, 24 h), to diminish EN-induced cAMP increase in HMEC-1 cells (Supplementary Figure S5), and by (ii) previous observations, such as the impairment of β2ADR function in asthmatic children who are exposed to mixtures of PAHs^16^, and the down-regulation of β2ADR in rat lung alveolar macrophages after chronic exposure to cigarette smoke^29^.

We reported in this study that short-term exposure to B(a)P, reduced β2ADR number at the cell surface (Fig. 3a). Mechanistically, this effect seems to essentially result from the internalization of β2ADR as no protein degradation was detected at this exposure time (Fig. 3b). However, further studies would be required to confirm this conclusion and to characterize the underlying molecular mechanisms. In this context, it might be interested to consider the phosphorylation of β2ADR by protein kinases, *e.g*. PKC, β-arrestin or β2-ARK^26^, as well as the implication of clathrin-coated vesicles known to interfere with agonist-induced β2-ADR internalization^30^.

We also reported that B(a)P can induce β2ADR down-regulation by enhancing receptor degradation at both mRNA and protein levels (Figs 3c  and 4f). It is worthy noting here that B(a)P-induced modulations of mRNAs encoding the β2ADR were of biphasic nature (Fig. 4a): a short-term exposure (up to 1 h) stimulates the transcription of β2ADR mRNA, whereas more prolonged exposure decreases β2ADR mRNA level (Fig. 4a) due to the reduction of β2ADR mRNA half-life associated to the acceleration of mRNAs degradation (Fig. 4f). Interestingly, similar biphasic modifications in β2ADR mRNA expression were also correlated to EN exposure in our cellular model (Fig. 4a), thus confirming previous observations^25^. Therefore, our results argue in favor of an agonist-like effect of PAHs on β2ADR transcripts. While the functional interpretation of the first increasing phase in the integral β2ADR response is still unclear, it is well documented that the late reduction of β2ADR expression could contribute to the receptor loss-of-function after agonist stimulation^31^.

To continue this work, it would be interesting to thoroughly study the molecular effectors contributing to PAH-induced biphasic regulation of β2ADR. Indeed, short activation of β2ADR by B(a)P increases intracellular cAMP concentration, via the activation of adenylyl cyclase^10^. We might speculate that this cAMP elevation could be sufficient to activate a PKA-dependent CREB pathway, thus inducing the transcription of genes containing in their promoter the cAMP responsive element (CRE), such as β2ADR gene. This β2ADR/cAMP/PKA/CREB/CRE pathway is known to exert a positive transcriptional autoregulation^26^, thereby likely contributing to the early increase in β2ADR mRNA expression observed after receptor activation by B(a)P. However, when receptor stimulation by B(a)P is chronic, desensitization processes would be initiated. In this case, it could be observed the cAMP-dependant activation of β-ADR mRNA-binding protein (β-ARB) or of A/U rich mRNA binding factor (AUF1) that causes the β2ADR mRNA decay. This pathway is usually associated with receptor down regulation observed during desensitization process^32, 33^. Beside these G protein-dependent mechanisms, the β-arrestin-dependent, G protein-independent pathway may also explain the observed effects on β2ADR mRNA. This β-arrestin-dependent pathway is known to create a link between β2ADR activation and many cellular pathways including the recruitment of c-SRC kinase and the activation of the MAP kinase signaling network, both known to be implicated in the β2ADR desensitization^34–37^. Whether B(a)P, by its capacity to activate β2ADR, could trigger this β-arrestin-dependent desensitization pathways will therefore be worth considering.

Interestingly, beside its role in regulating β2ADR desensitization, c-SRC kinase is also known to be associated to the cytosolic AhR multiprotein complex. Binding of AhR ligands, such as B(a)P, to this complex leads to dissociation, activation and translocation to plasma membrane of c-SRC, which may then represent a molecular link between AhR and membrane proteins (including GPCRs) as an element of the non-genomic pathway of AhR^38^. However, c-SRC kinase is not the only one potential link between the AhR and GPCR pathways; cAMP and intracellular calcium signals are also known to play important roles in both AhR- and GPCR-depending function and regulation. Indeed, AhR ligands as TCDD and B(a)P, are able to increase intracytosolic calcium concentration and/or the production of cAMP^10, 39^. Both Ca^2+^ and cAMP represent classical second messengers associated to GPCR activation and desensitization, as known for β2ADR, and also exhibit the capacity to influence the nuclear translocation of AhR^40, 41^. In this context, the question that arises next is: does it exist a reciprocal regulation between AhR and GPCRs pathways? In line with this, it is noteworthy that similar crosstalk has already been demonstrated between GPCRs and other nuclear receptors such as the glucocorticoid receptor^42, 43^.

In conclusion, we showed that exposure to B(a)P triggered β2ADR desensitization and consequently reduced cellular capacities to react to the stimulation by the physiological ligand EN. Keeping in mind that functional β2ADR signaling is required for normal cardiovascular and pulmonary^44–46^ as well as for the neurodevelopment^47–49^, and that humans are continuously exposed to PAHs, our work therefore suggests that the interaction between PAHs and β2ADR might contribute to PAHs toxicity, not only by eliciting cellular responses such as calcium signal or CXCL8 release, but also by disrupting of cardiovascular, pulmonary and neurological physiological homeostasis. These results also suggest that even if the majority of cellular effects of PAHs are attributed to AhR activation, the implication of other signaling pathways, such as that of β2ADR, in PAH-related toxicity can no longer be discarded.

## Material and Methods

### Chemical and Reagents

B(a)P, EN, ICI 118.551, salbutamol, propranolol and actinomycin D were provided by Sigma-Aldrich (Saint-Quentin Fallavier, France). Rabbit monoclonal antibody anti-β2ADR and control antibody were obtained from Santa Cruz Biotechnology (Santa Cruz, CA). All other compounds were commercial products of the highest purity available. Chemicals were used as stock solutions in DMSO; the final concentration of this solvent in culture medium was always <0.2% (v/v), and control cultures received the same dose of vehicle as exposed cultures.

### Cell Culture

Human microvascular endothelial cells HMEC-1, obtained from the Center for Disease Control and Prevention (Atlanta, GA), were routinely maintained in MDCB-131 medium containing epidermal growth factor (10 ng/mL), hydrocortisone (1 µg/mL), glutamine (10 mM), penicillin (50 units/ml) and streptomycin (50 µg/ml) and supplemented with 10% fetal calf serum. Before each treatment, cells were cultured over night in medium without calf serum, for serum catecholamines weaning.

### Intracellular cAMP Measurements

Cellular concentration of cAMP (pmol/well) was quantified by the chemiluminescent immunoassay cAMP-Screen^TM^ System (Applied Biosystems, Foster City, CA), according to the manufacturer’s instructions.

### RNA Isolation and Analysis

Total RNA was isolated from cells using the TRIzol method (Invitrogen); it was then subjected to reverse transcription real-time quantitative PCR (RT-qPCR) analysis, as previously described^50^. Primers were as follows: β2ADR-forward: 5′-TTCCTTCCTACACCCTTGGA-3′; β2ADR-reverse: 5′-AGACTTTGCTCGGGAAAACA-3′; NR4A1-forward: 5′GTTCTCTGGAGGTCATCCGCA-3′; NR4A1-reverse: 5′-GACGGGACCTTGAGAAGGCCA-3′; NR4A2-forward: 5′-TATTCCAGGTTCCAGGCGAA-3′; NR4A2-reverse: 5′-GCTAATCGAAGGACAAACAG-3′; NR4A3-forward: 5′-CCAAGCCTTAGCCTGCCTGTC-3′; NR4A3-reverse: 5′-AGCCTGTCCCTTACTCTGGTGG-3′; 18S-forward: 5′-CGCCGCTAGAGGTGAAATTC-3′; 18S-reverse: 5′-TTGGCAAATGCTTTCGCT-3′. The specificity of each gene amplification was verified at the end of qPCR reactions through analysis of dissociation curves of the qPCR products. Amplification curves were analyzed with ABI Prism 7000 SDS software using the comparative cycle threshold method. Relative quantification of the steady-state target mRNA levels was calculated after normalization of the total amount of tested cDNA to an 18S RNA endogenous reference.

### mRNA Half-life Determination

We have measured mRNA half-life using a method blocking cellular transcription with actinomycin D^51^. After 1 h of treatment by EN or B(a)P, actinomycin D (5 µg/ml) was added to culture medium to inhibit *de novo* mRNA synthesis; then treatments were stopped after 0, 30, 60, 90 or 150 min, and mRNA levels analyzed after extraction, by real time-PCR. The half-life of mRNA was calculated, after linear regression, from the slope of a semilogarithmic plot of mRNA level.

### Immunolocalization

Cells were fixed with cold solution of paraformaldehyde (4% in PBS) for 30 min (on ice). After removal of paraformaldehyde, cells were washed with PBS and incubated in PBS containing 2% BSA for 1 h at room temperature. The medium was aspirated and replaced with blocking medium containing primary antibody (isotype control or antibody against β2ADR). After 2 h of incubation at room temperature and three washes with cold PBS, the primary antibody was detected with goat fluorescein-labeled IgG (FITC). Nuclei were stained by 4′,6-diamidino-2-phenylindole (DAPI), for 5 min at room temperature. Images were then acquired with a fluorescence microscope adapted to a high resolution AxioCam camera (Carl Zeiss, Canada).

### Immunoblotting Analysis

Immunoblotting was performed on total cellular extracts as previously described^52^. Briefly, protein samples (40 µg) were subjected to electrophoresis in a 10% acrylamide gel and electrophoretically transferred to a nitrocellulose membrane (Bio-Rad). After blocking with Tris-buffered saline containing 4% bovine serum albumin and 0.1% Tween 20 at room temperature, membranes were incubated with specific primary antibody overnight at 4 °C and, subsequently, with appropriate horseradish peroxidase-conjugated secondary antibody for 1 h. Immunolabeled proteins were finally visualized by chemiluminescence using the LAS-3000 analyzer (Fujifilm). Image processing was performed using Multi Gauge software (Fujifilm).

### Statistical Analysis

Results are presented as means ± S.D. They were statistically analyzed with student’s *t* test. *p* < 0.05 was considered statistically significant.

## Electronic supplementary material

Supplementary information
