# Supplementary material for: Benzo(a)pyrene triggers desensitization of β2-adrenergic pathway
Source: Sci Rep. 2017 Jun 12;7:3262. doi: 10.1038/s41598-017-03646-4 (PMC5468268; doi:10.1038/s41598-017-03646-4)
Supplement: Supplementary file 1 — Supplementary information [file 41598_2017_3646_MOESM1_ESM.pdf]

Supplementary Figures

Benzo(a)pyrene triggers desensitization of β2-adrenergic pathway

Abdullah Mayati[1,2], Normand Podechard[1,2], Manuelle Rineau[1], Lydie Sparfel[1,2], Dominique Lagadic-Gossmann[1,2], Olivier Fardel[1,2,3], and Eric Le Ferrec[1,2].

[1] Inserm U1085, Institut de Recherche en Santé, Environnement, Travail, Rennes, France;
[2] Université de Rennes 1, Faculté des Sciences pharmaceutiques et biologiques, Biosit UMS3080, 35043 Rennes Cédex, France;
[3] Pôle Biologie, Centre Hospitalier Universitaire, 2 rue Henri Le Guilloux, 35033, Rennes, France.

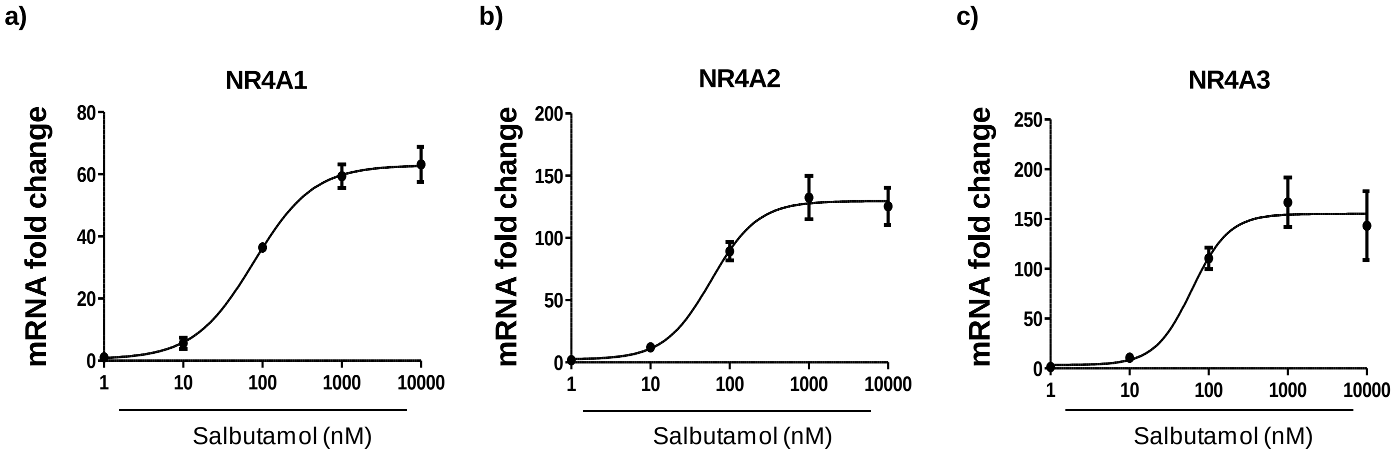

Fig. S1: Induction of NR4As expression triggered by Salbutamol. HMEC-1 cells were either untreated or exposed to indicated concentrations of salbutamol (selective β2-agonist) for 1 h. mRNA expressions of β2ADR-target genes, NR4A1 (a), NR4A2 (b) and NR4A3 (c) were next determined by RT-qPCR. Data are expressed relatively to mRNA levels found in untreated cells, arbitrarily set to 1 unit, and are the means ± S.D of at least three independent assays.

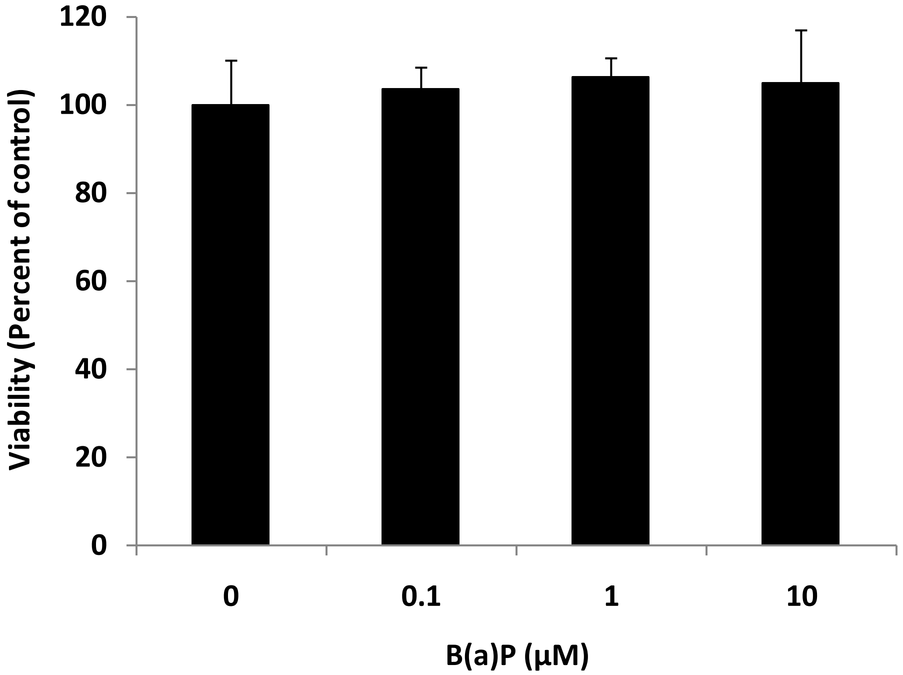

Fig. S2 : B(a)P treatment does not affect cell viability whatever the dose used. Cytotoxic effect of B(a)P treatment toward HMEC-1 cells was assessed using the 3-(4–5-dimethylthiazol-2-yl)- 2,5-diphenyltetrazolium bromide (MTT) colorimetric assay. Briefly, cells were seeded in 96-well plates. After 3 days of culture, cells were cultured overnight in medium without calf serum (for serum catecholamines weaning) and then exposed for 24 h to various concentration of B(a)P. Cells were then incubated with 100 µl of MTT solution (0.5 mg/ml) for 2 h at 37°C in a 5% CO_2 atmosphere. Medium was thereafter discarded and replaced by 100 µl of DMSO. Blue formazan formed products were further quantified by their absorbance at 540 nm using SPECTROstar Nano (BMG Labtech, Ortenberg, Germany).

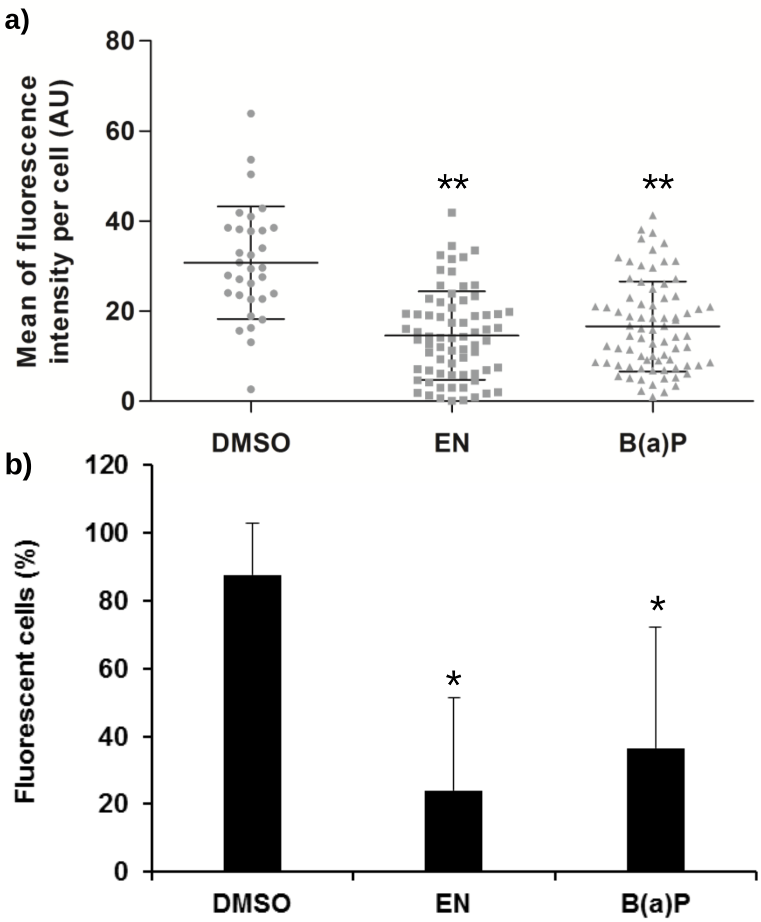

Fig. S3: B(a)P triggered an early reduction of β2ADR expression at cell membrane.

HMEC-1 cells were exposed to 10 µM epinephrine (EN), 1 µM B(a)P or vehicle (DMSO) for 45 min. From images of β2ADR expression at cell membrane obtained after immunolocalization, fluorescence signals were analyzed by quantification on ImageJ software. (a) Means of fluorescence intensity (MFI) for each analyzed cells are presented as a scatter plot and global means ± S.D are in black line. Data shown are from three independent assays. (b) Based on MFI, the percentage of positives fluorescent cells per experiment was determined (Cell was considered to be positive when its MFI was higher than the 25th percentile of MFI values observed in the pool of control cells from all experiments *i.e.* 22.66 AU), data presented are means ± S.D from three independent assays. *, $p < 0.05$; **, $p < 0.01$ when compared to untreated cells.

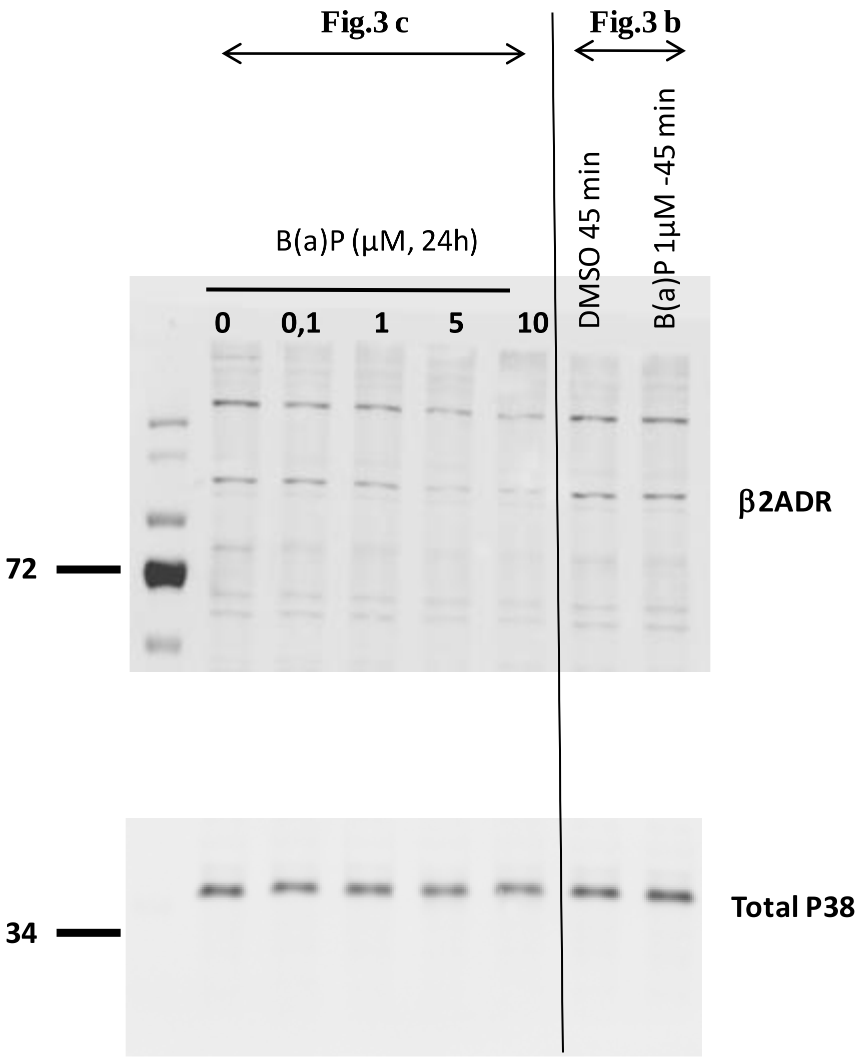

Fig. S4 : western blots , whole membranes used for Fig.3 c and b

HMEC-1 cells were exposed to 1 µM B(a)P or vehicle (DMSO) for 45 min and then β2ADR or total p38 protein content was then determined by Western-blotting. Protein samples (40 µg) were subjected to electrophoresis in a 10% acrylamide gel and electrophoretically transferred to a nitrocellulose membrane (Bio-Rad). After blocking with Tris-buffered saline containing 4% bovine serum albumin and 0.1% Tween 20 at room temperature, membranes were incubated with specific primary antibody overnight at 4 °C and, subsequently, with appropriate horseradish peroxidase-conjugated secondary antibody for 1 h. Immunolabeled proteins were finally visualized by chemiluminescence using the LAS-3000 analyzer (Fujifilm). Image processing was performed using Multi Gauge software (Fujifilm)

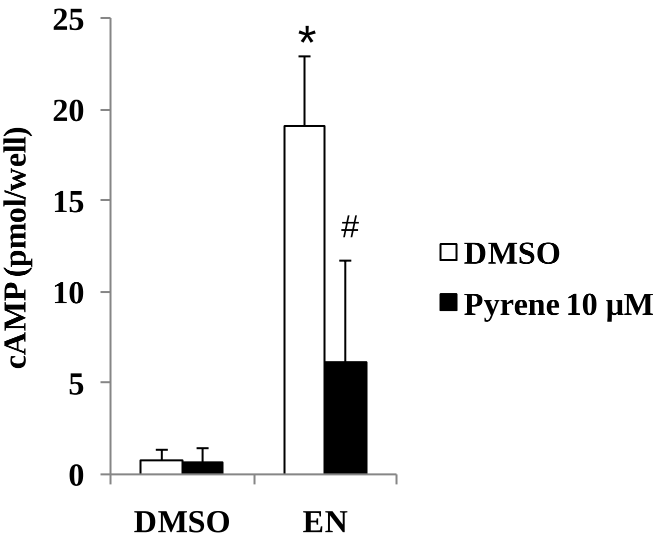

Fig. S5: Pyrene decreased the epinephrine (EN) effect on intracellular cAMP level. As for B(a)P (see Fig. 2), HMEC-1 cells were exposed to 10 µM Pyrene or vehicule (DMSO) for 24 h, and then co-exposed or not to 10 µM EN for 10 min. cAMP levels were next determined, as described in "Experimental Procedures". Data are the means ± S.D of three independent assays. *, $p < 0.05$ when compared to untreated cells ; #, $p < 0.05$ when compared to EN-treated counterparts.
